# Editorial: Cancer Care Delivery and Women’s Health: Beyond the Patient and Provider Relationship

**DOI:** 10.3389/fonc.2017.00213

**Published:** 2017-09-14

**Authors:** Sarah M. Temkin

**Affiliations:** ^1^Virginia Commonwealth University, Richmond, VA, United States

**Keywords:** cancer care delivery, women’s health, gynecologic cancer, breast cancer, health systems

**Editorial on the Research Topic**

**Cancer Care Delivery and Women’s Health: Beyond the Patient and Provider Relationship**

The number of cancer patients and survivors worldwide continues to grow as a result of our growing and aging population. In 2013, an Institute of Medicine report detailed a crisis of the cancer care delivery system resulting in larger numbers of cancer patients combined with increasingly complex treatments and rising costs associated with health care (1). Since that time advances in genomics and a call for precision medicine have augmented these concerns and our expenditures on cancer care have continued to rise.

Multiple factors within the health-care system impact the experience of the cancer patient and oncology provider. Women with cancer are often the primary social support of their family creating unique social impediments for the families of patients. Additionally, part of a diagnosis of breast or gynecologic malignancy may include a perceived loss of “womanhood” and related body image concerns (2). Historical inequality, cultural perceptions, and attitudes and implicit bias impact the way that female cancer patients interact with the health-care system and may complicate shared decision-making and generate psychosocial barriers to quality care delivery. The multilevel interventions needed to advance the care and experience of the breast and gynecologic cancer patient are, therefore, distinct. In this issue of Frontiers in Oncology, Women’s Health, we explore the specific challenges of the cancer care delivery system as it relates to the care of women with breast and gynecologic cancer.

Cancer care delivery refers to the multiple layers of the health-care system that interact to affect outcomes for patients diagnosed with malignancies and the quality of that care. These layers include but are not exclusive to the patient, her caregiver, the health-care team, the clinic or hospital, the health insurance system, pharmaceutical companies, and the government. Whereas cancer care of the 20th century primarily revolved around the oncologist–patient relationship, the scope of care for the cancer patient and survivor has grown significantly. Oncologic outcomes can be negatively impacted by the stress of navigating the complex structures of the health-care system (3, 4). The network of cancer care now includes multiple additional practitioners that the patient is in direct contact with (physical therapists, nutritionist, wound care nurse, etc.); practitioners that patient will never see (radiologists, pathologists, etc.); and countless ancillary staff members (tumor registrar, health insurance specialists, electronic medical records information technologist, etc). New subfields related to oncology (supportive care, onco-dermatology, etc.) have flourished. This intricate web of consultants has expanded to the point that patient navigators are now routinely employed within large cancer centers to ensure that the patient is able to find her way through the cancer care experience. Special populations such as the poor, the elderly, and minority women are at particular risk of getting lost within the system. Despite all this complexity, simple, inexpensive therapies such as collecting patient-reported outcomes or integrating palliative care into standard oncology practice that have demonstrated, statistically significant, clinical benefits are underutilized in clinical practice (5, 6).

Interdisciplinary teamwork is essential for all of these moving parts to function seamlessly, but teamwork and inter-team cooperation is not always incentivized by the health-care system (7). Shared decision-making becomes challenging when overlapping teams participate in the care of an individual patient without coordination and clear lines of communication. Novel oncology payment models, including accountable care organizations and pay for performance are being studied to improve a more collaborative approach to cancer care. The goal of innovative reimbursement strategies is to encourage all parts of this multifaceted system to work together while controlling cost (8).

Working toward a goal of a seamless patient experience within a system where all the moving parts work toward a common goal of best cancer care has spurred a new field of research—Cancer Care Delivery Research—which is defined by the National Cancer Institute as “how social factors, financing systems, organizational structures and processes, health technologies, and health-care provider, and individual behaviors affect cancer outcomes, access to and quality of care, cancer care costs, and the health and well-being of cancer patients and survivors.” This field of research extends upon quality improvement and focuses on multilevel interventions to improve and inform cancer care through modifications of the structures and processes of cancer care delivery to enhance the patient experience and optimize value (9). Standard measures of care quality are needed outcomes to be accurately reported. Big data—electronic health sets so large and complex that they are difficult to manage with traditional software—are essential to this charge (10). Big data have the potential to transform the way health-care systems use technologies to provide feedback to practitioners and expand the evidence base for quality care in near real time.

Ultimately reducing fragmentation, increasing coordination and accurately measuring outcomes within the cancer care delivery system has enormous potential to improve oncologic care for women with breast and gynecologic cancers by minimizing under- and over-treatment, reducing health-care disparities and improving the experience of cancer care for patients and caregivers.

## Author Contributions

The author is responsible for the design of this article, the research, and the writing.

## Conflict of Interest Statement

The author declares that the research was conducted in the absence of any commercial or financial relationships that could be construed as a potential conflict of interest. The reviewer, KS, and handling editor declared their shared affiliation.
